# Supplementary material for: Ancient genomes illuminate Eastern Arabian population history and adaptation against malaria
Source: Cell Genom. 2024 Feb 27;4(3):100507. doi: 10.1016/j.xgen.2024.100507 (PMC10943591; doi:10.1016/j.xgen.2024.100507)
Supplement: Supplementary file 1 [file mmc1.pdf]

Cell Genomics, Volume *4*

Supplemental information

Ancient genomes illuminate Eastern Arabian population history and adaptation against malaria

Rui Martiniano, Marc Haber, Mohamed A. Almarri, Valeria Mattiangeli, Mirte C.M. Kuijpers, Berenice Chamel, Emily M. Breslin, Judith Littleton, Salman Almahari, Fatima Aloraifi, Daniel G. Bradley, Pierre Lombard, and Richard Durbin

Supplementary Information

Ancient genomes illuminate Eastern Arabian population history and adaptation against malaria

Authors: Rui Martiniano, Marc Haber, Mohamed A. Almarri, Valeria Mattiangeli, Mirte C. M. Kuijpers, Berenice Chamel, Emily M. Breslin, Judith Littleton, Salman Almahari, Fatima Aloraifi, Daniel G. Bradley, Pierre Lombard, Richard Durbin

Supplementary Figures

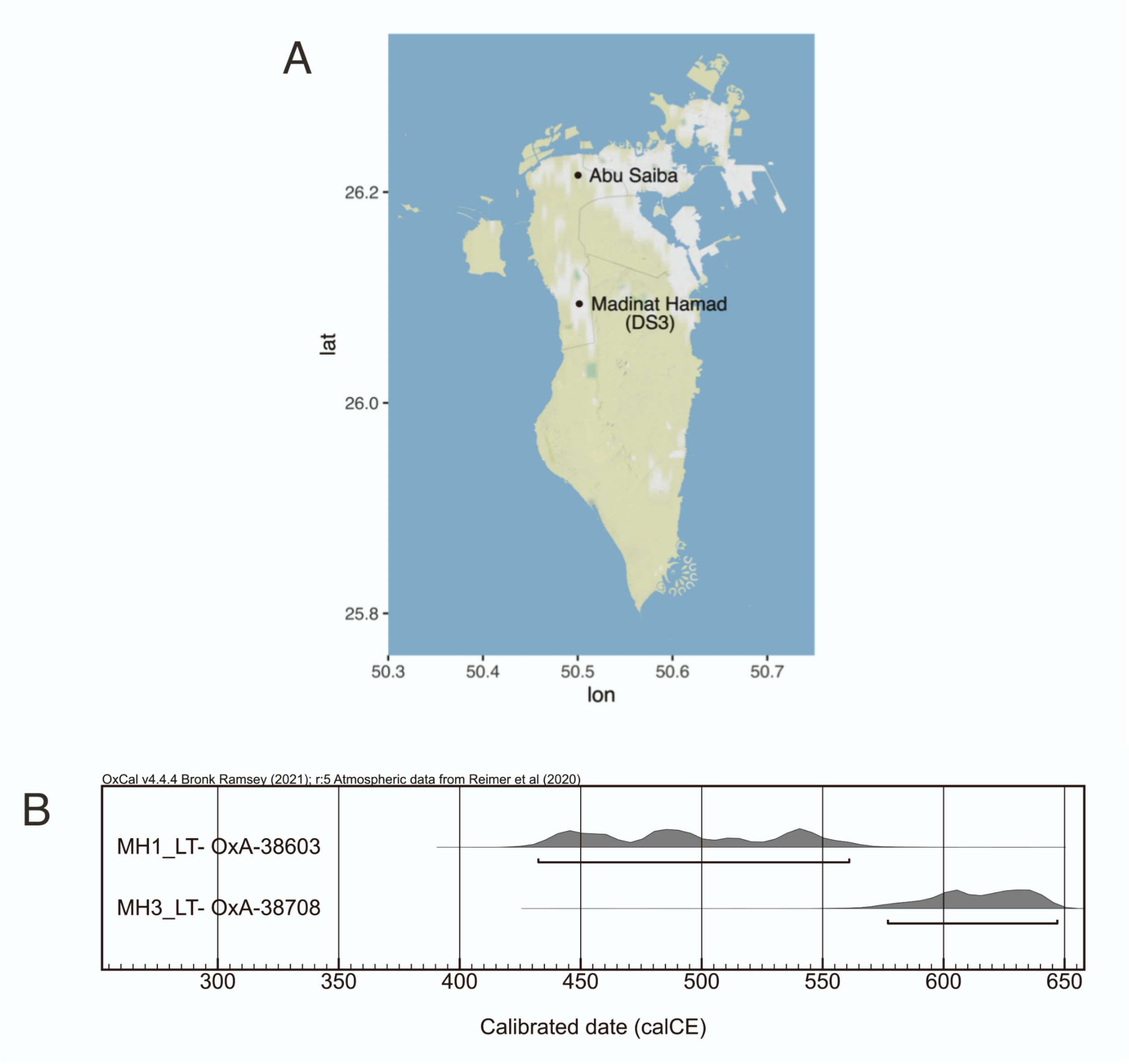

Figure S1 - Geographical and temporal information about the Bahrain Tylos samples, related to STAR Methods. A) Geographical locations of Abu Saiba and Madinat Hamad archaeological sites in Bahrain. B) Radiocarbon dating of two Bahrain samples, showing the density and 95.4% bars.

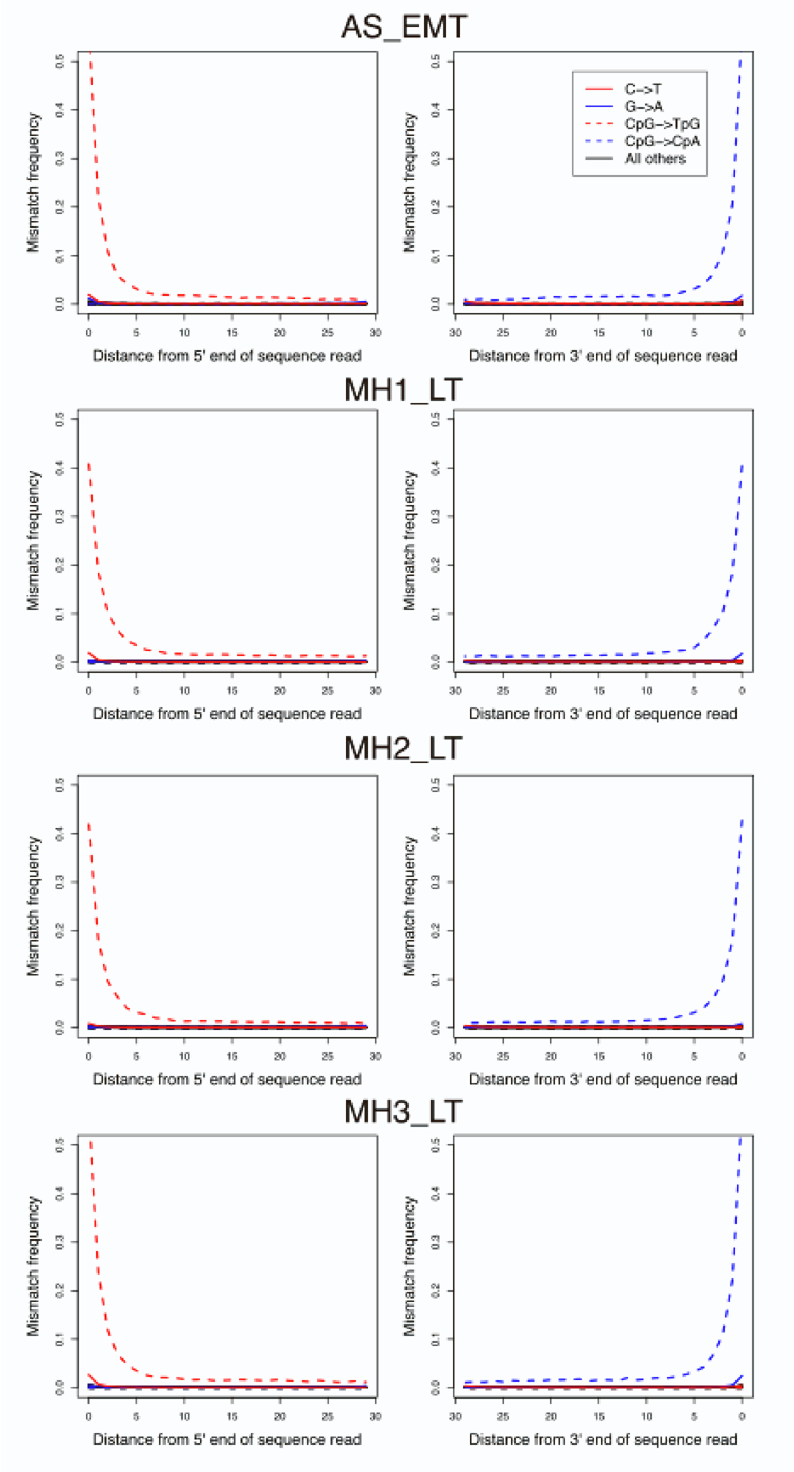

Figure S2 - Deamination patterns in the four ancient Bahrain samples, related to STAR Methods. As expected in enzymatically treated samples, deamination is still observable at CpG sites.

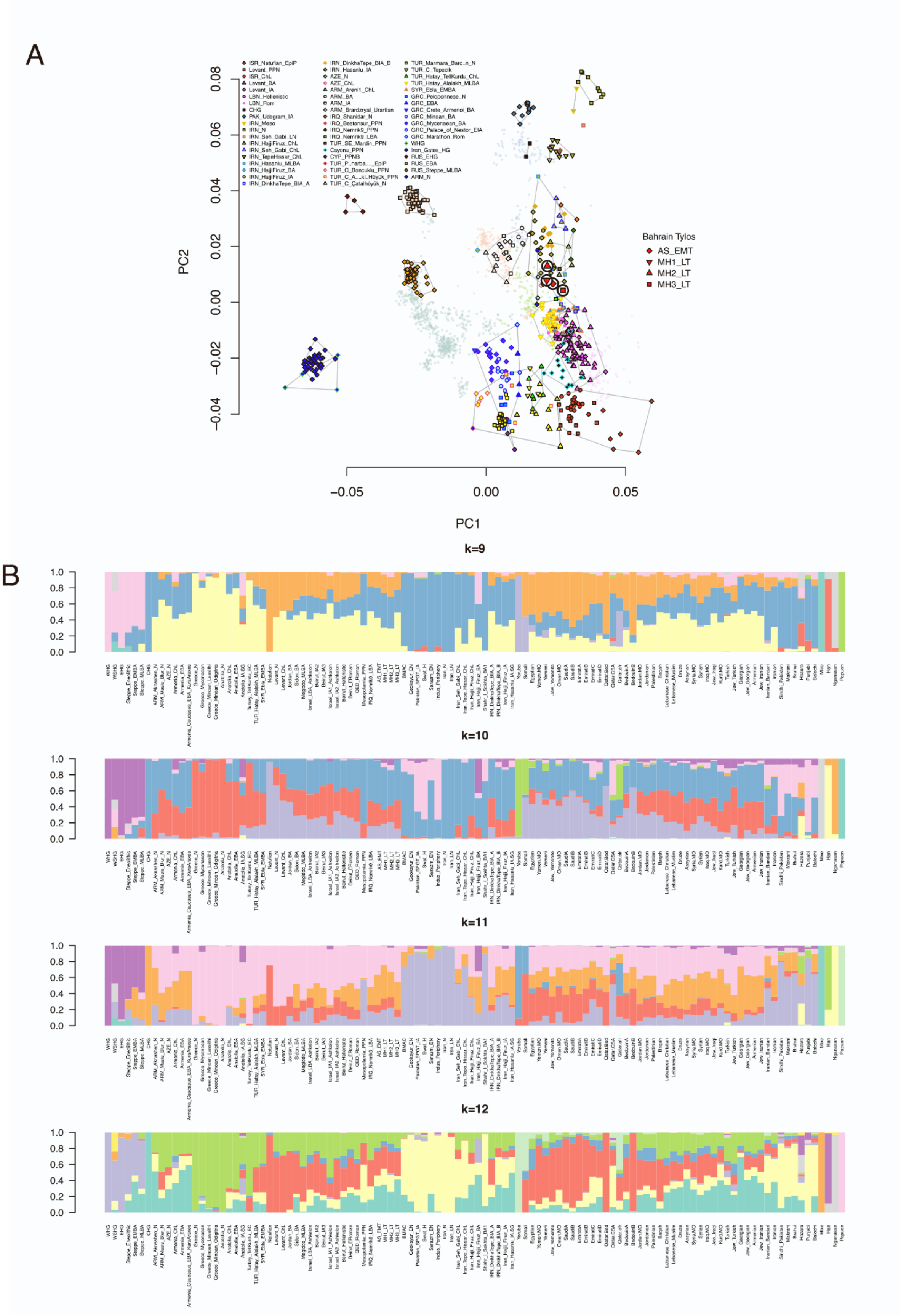
A
PC1
PC2
Bahrain Tylos
AS_EMT
MH1_LT
MH2_LT
MH3_LT
B
k=9
k=10
k=11
k=12

Figure S3 - Ancestry composition and population affinities of Bahrain Tylos samples, related to STAR Methods. A) Principal Component Analysis with 1,830 present-day and ancient Eurasians and 579,407 SNPs. Ancient samples are indicated with larger symbols as in the key. B) Dystruct analysis k=9 to k=12.

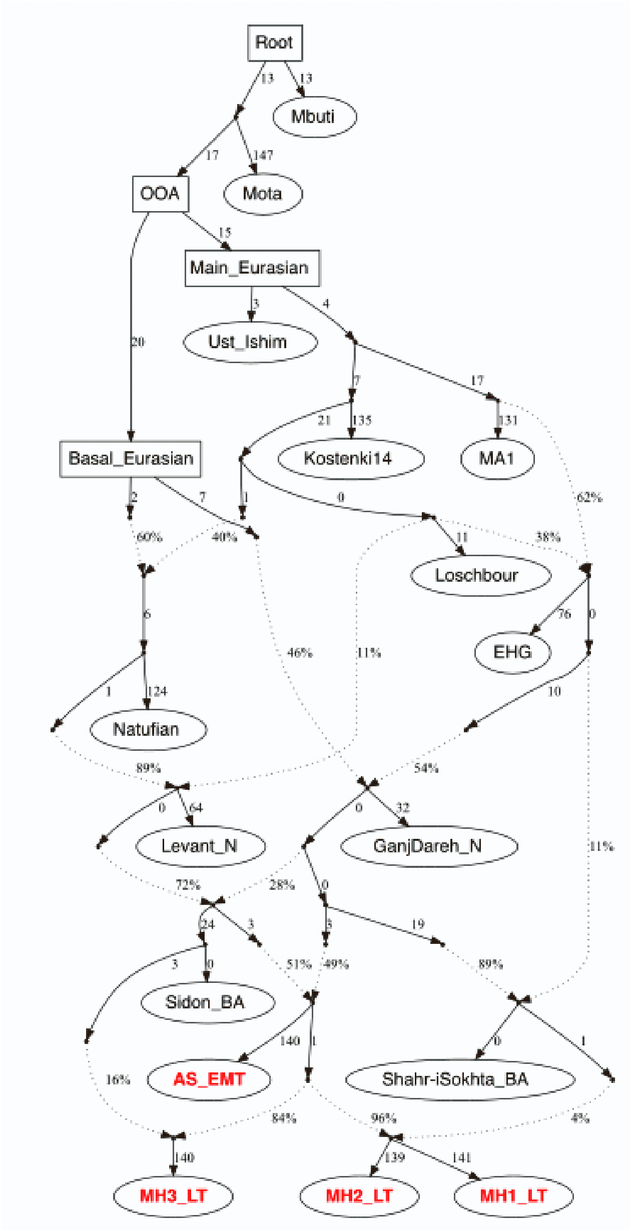

Figure S4 - Alternative model of Bahrain Tylos ancestry using qpGraph and IRN_Shahr-i Sokhta_BA instead of IRN_Hajji_Firuz_IA, related to STAR Methods.

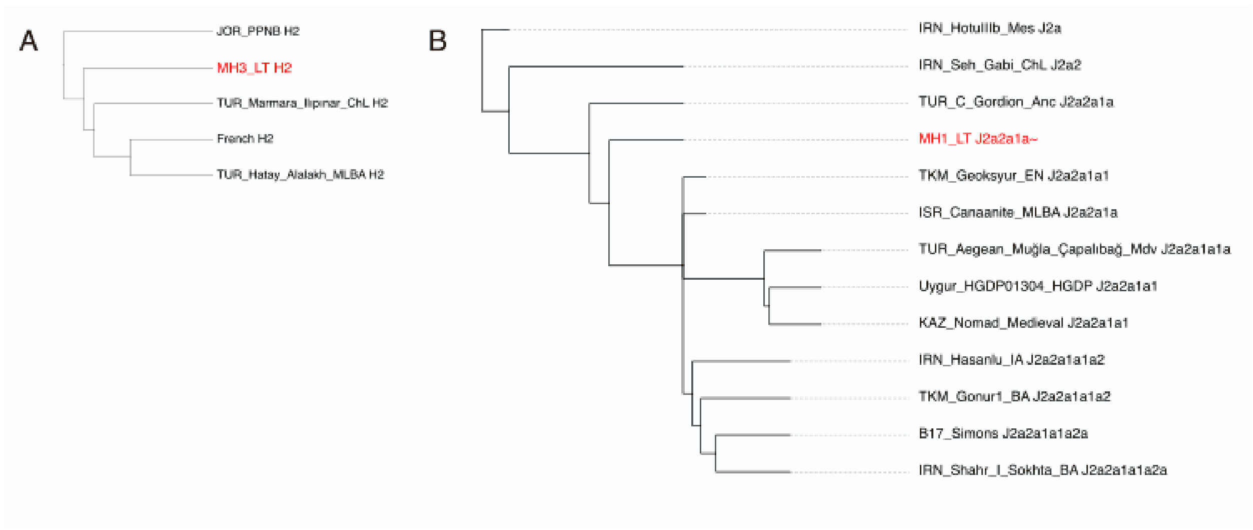

Figure S5 - pathPhynder placement of two Bahrain Late Tylos and other relevant ancient samples in a phylogeny of present-day and ancient Y-chromosome variation, related to STAR Methods. A) H2 Y-chromosome lineages. B) J2a Y-chromosome lineages.

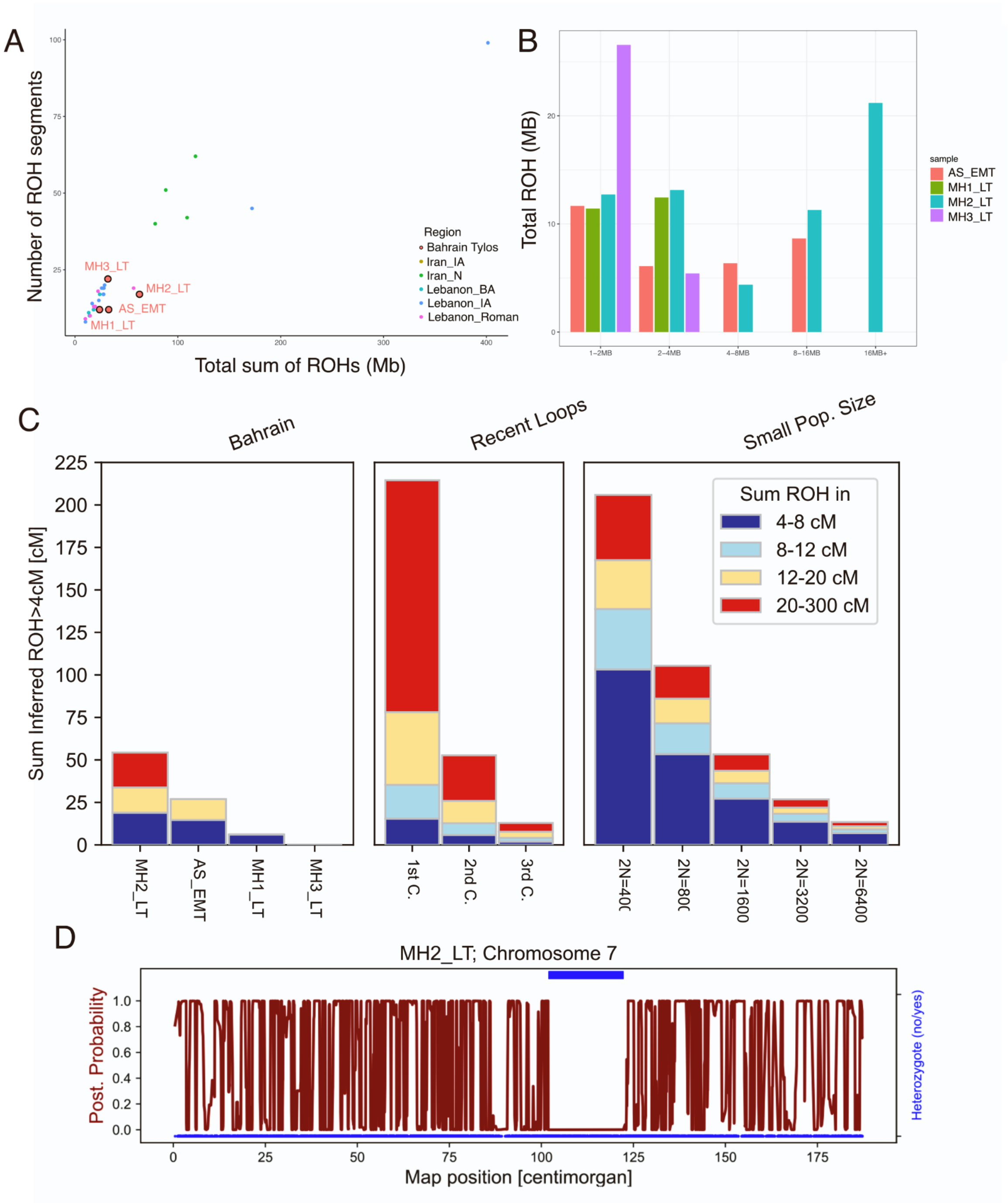

Figure S6 - Runs on homozygosity in Bahrain Tylos samples, related to STAR Methods. A) Tylos-period Bahrain ROH distribution compared to ancient Levantine and Iranian samples. B) ROH size distribution among Tylos-period Bahrainis. C) Sum of inferred ROH>4cM in the four Tylos period samples from Bahrain and simulated proportions of ROH sizes in recent loops and small population sizes for comparison. D) ~20.47 cM segment of homozygosity found in the chromosome 7 of sample MH2_LT.

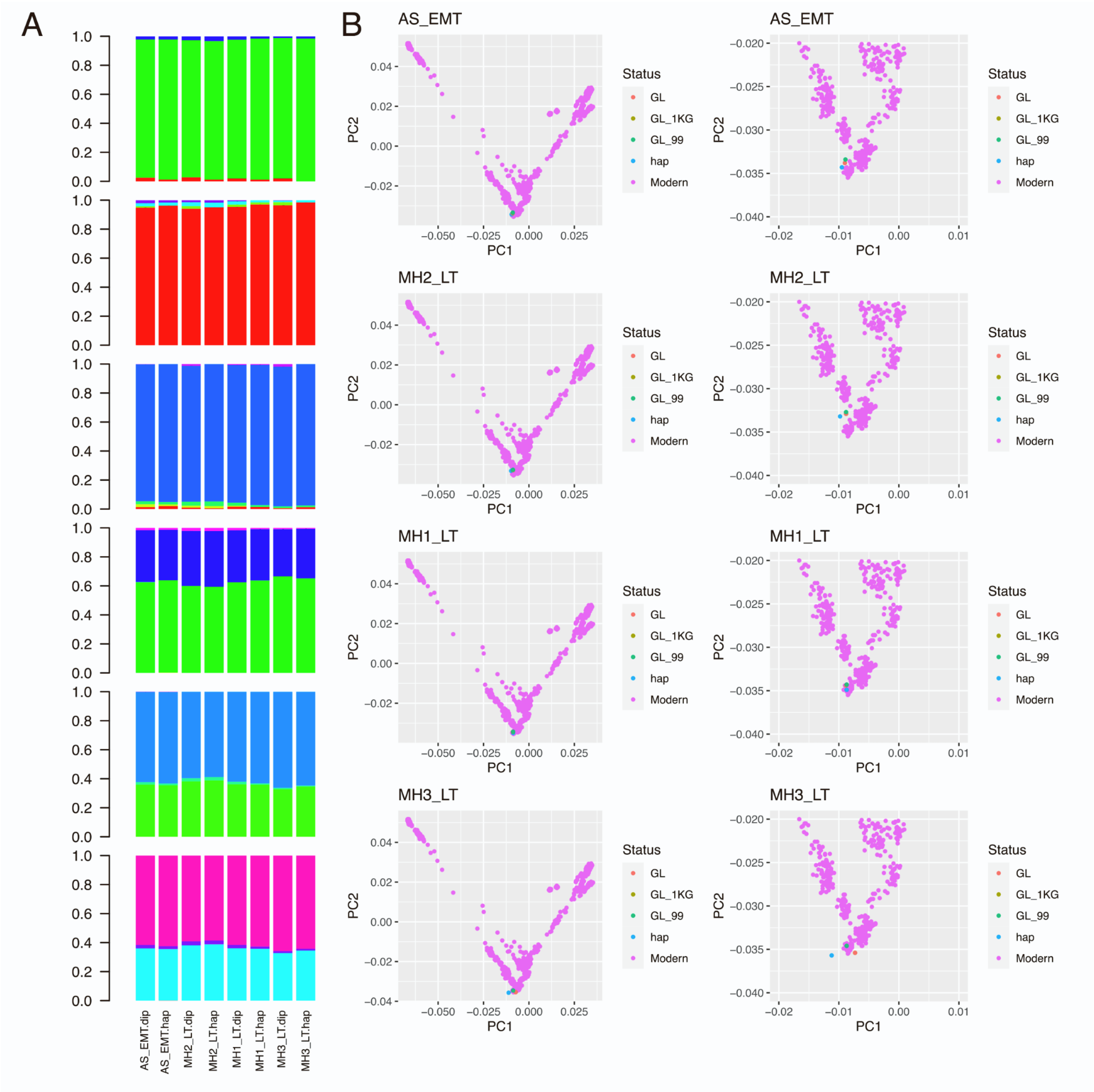

Figure S7 - Quality check of imputation, related to STAR Methods. A) PCA calculated on the HGDP and projecting pseudo-haploid calls with different phased diploid calls. B) Admixture comparing pseudo-haploid calls with final imputed calls (GLIMPSE using HGDP + Almarri et al., 2021 as reference, setting GP <99 to missing and re-imputing the missing sites using the 1000G high coverage reference panel). K3 to K8, samples run with HGDP dataset.
